# Norwegian and Swedish value sets for the EORTC QLU-C10D utility instrument

**DOI:** 10.1007/s11136-024-03824-8

**Published:** 2024-11-05

**Authors:** Gudrun Rohde, Jens Lehmann, Micha J. Pilz, Leslye Rojas-Concha, Bernhard Holzner, Madeleine T. King, Richard Norman, Georg Kemmler, On behalf of the EORTC Quality of Life Group

**Affiliations:** 1 Department of Health and Nursing, University of Agder, Kristiansand, Norway; 2 Department of Clinical Research, Sorlandet Hospital, Kristiansand, Norway; 3 Division of Psychiatry, Marie Curie Palliative Care Research Department, University College London, London, UK; 4 University Hospital of Psychiatry II, Medical University of Innsbruck, Innsbruck, Austria; 5 Palliative Care Research Unit, Department of Geriatrics and Palliative Medicine GP, Bispebjerg & Frederiksberg Hospital, University of Copenhagen, Copenhagen, Denmark; 6 School of Psychology, University of Sydney, Sydney, NSW Australia; 7 School of Public Health, Curtin University, Perth, WA Australia; 8 University Hospital of Psychiatry I, Medical University of Innsbruck, Innsbruck, Austria; 9 Faculty of Health and Sport Sciences, University of Agder, Box 522, 4602 Kristiansand, Norway

**Keywords:** Cancer, Utility, Quality of life, Discrete choice experiment, EORTC QLU-C10D

## Abstract

**Purpose:**

This study aimed to develop utility weights for the European Organization for Research and Treatment of Cancer (EORTC) QLU-C10D, a cancer-specific utility instrument, tailored to the Norwegian and Swedish populations. The utility weights are intended for use in the specific welfare contexts of Norway and Sweden to support more precise healthcare decision-making in cancer treatment and care.

**Methods:**

This cross-sectional study included 1019 Norwegian and 1048 Swedish participants representative in age and gender of the two general populations. Participants completed a discrete choice experiment involving 960 choice sets, each consisting of two EORTC QLU-C10D health states described by the instrument’s domains and the duration of each state. Utility weights were calculated using generalized estimation equation models, and non-monotonic levels were merged to ensure consistent valuation.

**Results:**

In the Norwegian participants, the largest utility decrements were seen for the domain of physical functioning (decrement of − 0.263 for highest level “very much”), followed by pain (decrement − 0.205 for level “very much”) and role functioning (− 0.139). Among the cancer-specific domains, nausea had the largest utility decrement (− 0.124). In the Swedish participants, the largest utility decrements were also observed for physical functioning (− 0.207 for the response “very much”), followed by pain (− 0.139), role functioning (− 0.133), and nausea (− 0.119). Emotional functioning also exhibited a sizable utility decrement (− 0.115).

**Conclusion:**

This study provides the first set of utility weights for the EORTC QLU-C10D specific to Norway and Sweden, reflecting the unique health preferences of these populations. The generated utility decrements can inform cost-utility analyses and optimize resource allocation in cancer care within the Norwegian and Swedish healthcare systems.

**Supplementary Information:**

The online version contains supplementary material available at 10.1007/s11136-024-03824-8.

## Background

The Scandinavian welfare system offers public health treatment and care that is free of charge for all inhabitants regardless of their health insurance and income. This system is unique [1]. As in all countries worldwide, the number of people in Scandinavia will continue to increase in the future and more people will be in need of public health care. This will include a larger population of older people living with chronic and life-threatening conditions, and fewer people to pay for public health care via taxes [2–4]. Recent developments in treatment, person-centred care, and targeted therapy imply that more people will survive and live longer despite the prevalence of diseases that were previously associated with a shorter lifespan, such as cancer [5].

These changes will present challenges for the current welfare model because of the new demographics, technologies, and medical treatments within cancer care [1]. Increasingly expensive cancer treatments and services will be required, and these will need to be prioritized when providing public treatment and care to those most in need [5]. Determining cost-effectiveness of services could help determine how to optimise health outcomes within available health budgets [6]. In a health-care system with limited resources, cost–utility analysis (CUA) is recommended during the decision-making process to identify which treatments, existing or new, and care should be prioritized and reimbursed to maximize societal health-care benefits. CUA is a commonly used economic evaluation technique that involves cost–utility calculations, in which the difference in total costs between two or more interventions is compared with the difference in utility expressed as quality-adjusted years (QALYs) [7]. A QALY represents the combination of a person’s quantity of life and quality of life; one year with an optimal Health-Related Quality of Life (HRQOL) equals to one QALY [7].

Generic utility measures [8], such as the EuroQoL-5 Dimensions (EQ-5D) [9] and the Short Form 6 Dimension (SF-6D) (based on the SF-36 Health Survey) [10], have been validated and are used widely in Norway [11, 12] and Sweden [11, 13]. While generic utility measures are useful for comparisons across diseases [8], they do not include the full range of the HRQOL domains or symptoms that may be relevant for specific diseases [14, 15]. For cancer patients, a cancer-specific utility instrument has been developed based on the widely used European Organization for Research and Treatment in Cancer quality of life core module (EORTC QLQ-C30) [16], the EORTC Quality of Life Utility Core 10 Dimensions (EORTC QLU-10D) [17]. Five of these dimensions are generic aspects of functioning, and five are symptoms commonly experienced by cancer patients. Because of its backward compatibility with the EORTC QLQ-C30, EORTC QLU-C10D health utilities can also be calculated from EORTC QLQ-C30 data for any countries for which the utility weights are available.

Different countries have different approaches to decision-making about cancer health care funding; however, utility measures are often used [18, 19]. To calculate utilities for the EORTC QLU-C10D, national utility weights for each dimension are needed. The utility weights are based on the preferences of the general population [20] and may differ between countries and cultures [21]. EORTC QLU-C10D utility weights have been published for several countries including Australia [22], Canada [23], the United Kingdom (UK) [24], United States [25], Germany [26], France [27], Netherlands [28], Spain [29], Austria, Italy, and Poland [23], and Denmark [30]. However, such utility weights are lacking for Norway and Sweden.

The aim of the present study was to provide Norwegian and Swedish utility weights for the EORTC QLU-C10D using a discrete choice experiment (DCE) in the general Norwegian and Swedish populations.

## Methods

### Study design and populations

We relied on the previous methodology of King et al.[22]. The elicitation of EORTC QLU-C10D utility values relies on the assessment of trade-offs in health states using DCEs. This has been reported for various countries [22–31]. Here, we determined the EORTC QLU-C10D utility weights for Norway and Sweden. This cross-sectional study includes responses from a general public online research panel for the countries Norway and Sweden.

Assessments took place from 10 October to 29 November 2022 and were self-completed by participants from any place in the respective country. Participants were recruited via a third-party provider online panel (https://surveyengine.com/) and received a small payment for completing the assessment. We used quota sampling (by age and gender) in both general population samples to achieve representativeness for these two variables in each country. The initial eligibility criteria were sex (male, female) and aged between 18 and 80 years. Panel members who opted into the survey completed the introduction and disclosure sections, and those who consented progressed to the age and sex questions. Those who were younger than 18 years, did not declare sex male or female, fell within an age and sex quota that was already filled, or had entered the survey via a mobile (cell) phone were deemed ineligible for further participation and did not receive the compensation*.*

All participants provided electronic consent to participate, and the study was approved by the Ethics Committee of the Medical University of Innsbruck (approval number 1079/2023).

### Survey content

**Fig. 1:**
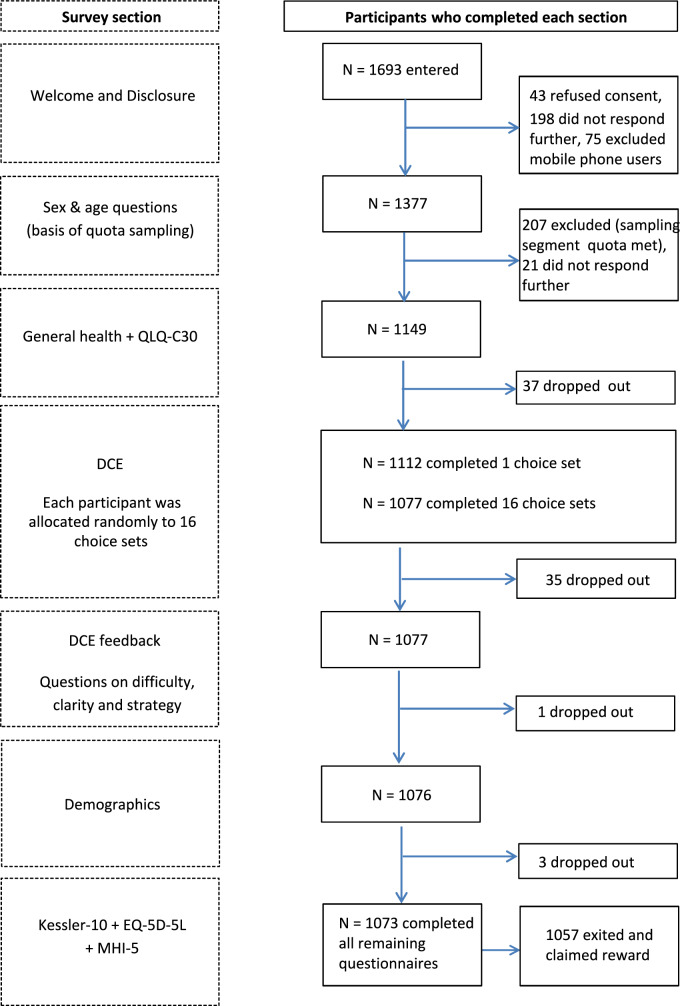

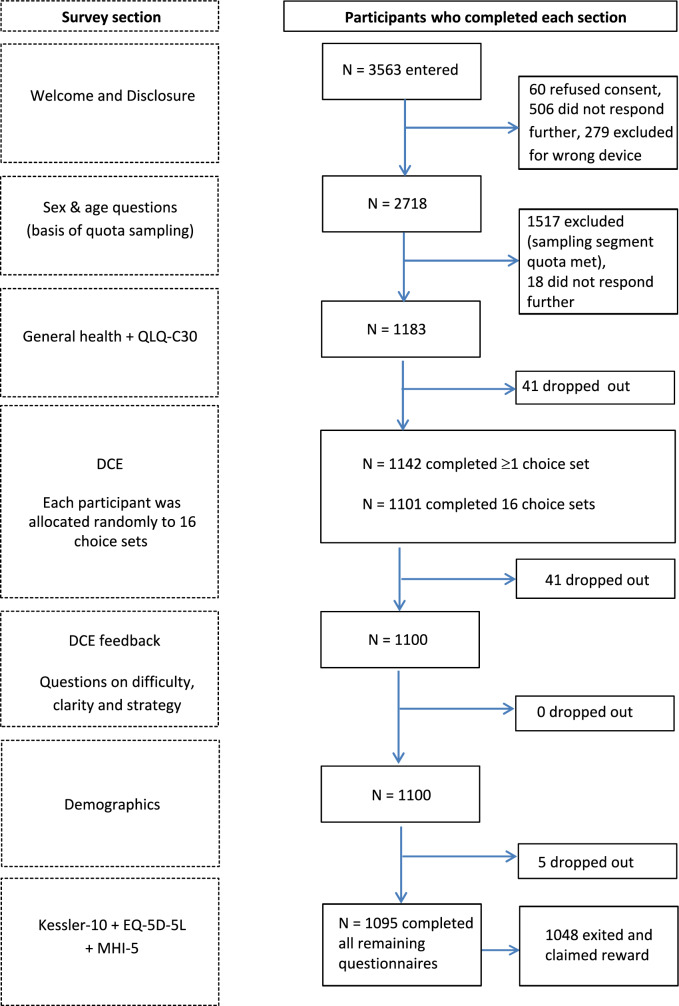
**a** Norwegian Respondent flow and sample sizes for each component of the valuation survey. **b** Swedish Respondent flow and sample sizes for each component of the valuation survey

Participants provided information on socio-demographic data and clinical data (chronic diseases) and completed the EORTC QLQ-C30, the DCE to establish Norwegian and Swedish utility weights, the EQ-5D-5L [32], and the Kessler psychological distress scale (K10 [33]). Their feedback on the applicability and difficulty in completing the DCEs was also assessed. We do not report the EQ-5D-5L or the K10 data in this paper. Figure 1a and b shows the survey components and the order in which they were presented.

### EORTC QLQ-C30 and EORTC QLU-C10D

The EORTC QLU-C10D is a utility-scoring algorithm for the EORTC QLQ-C30 that was developed by the EORTC and the Multi-Attribute Utility in Cancer (MAUCa) Consortium [17, 20, 22]. The EORTC QLQ-C30 is a 30-item cancer-specific questionnaire that assesses functional health (physical functioning, role functioning, social functioning, emotional functioning, cognitive functioning), symptom burden (fatigue, pain, nausea/vomiting, sleep disturbances, dyspnoea, appetite loss, constipation, diarrhoea, financial impact), and global health or quality of life [16]. For the EORTC QLU-C10D, 10 key domains from the EORTC QLQ-C30 were selected: physical functioning, role functioning, social functioning, emotional functioning, pain, fatigue, sleep disturbances, appetite loss, nausea, and bowel problems (which merges the constipation and diarrhoea scales from the EORTC QLQ-C30). Items are scored on a four-point Likert scale to assess the severity levels from “not at all” to “very much”. The EORTC QLU-C10D describes a total of 4^10^ = 1,048,576 different health states [17]. The classification system of the EORTC QLU-C10D is shown in Supplementary Table 1. The robustness of the EORTC QLU-C10D valuation methodology and its psychometric properties, such as test–retest reliability, have been demonstrated in recent studies [20, 34–36].

### Discrete choice experiments

Utilities were elicited using DCE methodology, and the DCEs were performed analogously to the methodology of King et al. [20, 22]. More detailed information on the DCE is given in Supplementary Material Fig. 1. Participants were randomly assigned to complete 16 of a total of 960 possible choice sets. For each choice set, participants were instructed to select the preferred scenario. Scenarios described unique health states representing different severity ratings across the 10 domains of the EORTC QLU-C10D. In addition, each scenario was assigned a specific survival time to be lived in this state of health (levels: 1, 2, 5, or 10 years; “You live in this state for X years, and then die”). To keep participant burden acceptable, the scenarios within a choice set differed in only five of the 11 aspects (10 QLU-10D domains and survival time). The differing aspects were highlighted to increase their visibility for participants [20].

### Statistical analysis

Descriptive statistics are reported for the sample characteristics including demographic and clinical information. Chi-square tests were used to compare the sample characteristics of gender, age, and education with national census data [37, 38]. To calculate utility weights, we followed an established procedure [39] that was also used in previous studies on the EORTC QLU-C10D [22–24, 26–31]. The utility (U) of an option “j” in the choice set “s” for a respondent “i” can be described by the following formula:

$$ Uisj = \alpha TIME_{isj} + \beta X^{\prime}_{isj} TIME_{isj} + \varepsilon_{isj} $$

where *TIME*_*isj*_ is the survival time in option *j* and *X′*_*isj*_ is a set of dummies related to the levels of the corresponding health state. Errors (*ε*_*isj*_) are assumed to be independent and identically normally distributed [22, 39]. Generalized estimation equation models with first-order autoregressive covariance structure were used for parameter estimation *(α, β*) to adjust for correlated observations within respondents [26]. Our previous studies showed that these models produce estimators that are comparable to conditional logit cluster sandwich estimators [29]. As in previous valuation studies, estimates were converted into utility decrements based on the ratio of health state parameters (*β*) and the time coefficient (*α*) to reflect the trade-off between HRQOL and survival. Finally, as in previous valuation studies, we ran an additional analysis to correct for non-monotonic levels in the EORTC QLU-C10D utility decrements; i.e., for domains in which the severity level did not show an increasing decrement in utility weight. To do this, we combined non-monotonic levels and calculated the model with a reduced number of estimates in β.

Analyses were performed using IBM SPSS Statistics (version 27.0; IBM Corp., Armonk, NY, USA).

### Data quality

We also performed the following data quality checks. We determined the percentage of participants who always chose the same response option in each of the 16 scenarios of the DCE (always “A” or always “B”); smaller percentages of these cases indicated high data quality. To determine whether DCE results were affected by the speed of completing the survey, we removed the 10% fastest respondents and compared the utility decrements of the remaining 90% with those of the entire sample by determining the mean absolute difference (MAD) between the two sets of utility decrements.

## Results

### Participant flow

An overview of the participant flows for Norway and Sweden are shown in Fig. 1a and b, respectively. A total of 22.499 persons were invited via email to participate in the survey. In Norway, 1696 persons entered the survey, and 307 were excluded because of quota sampling (i.e., they were not within the correct age or gender stratum). Of the remaining 1489 persons, 1019 (68.4%) completed the survey. In Sweden, 3563 persons started the survey, and 1517 were excluded for not being in the correct quota. Of the remaining 2046 persons, 1048 (51.2%) completed the survey.

### Socio-demographics and representativeness of the sample

The socio-demographic and clinical characteristics, and a comparison of these between the sample and census data for the general population are shown in Table 1. Because of quota sampling, the distributions of age and gender in the sample matched well with those in the population. Among both the Norwegian and Swedish participants, a significantly larger percentage of participants were highly educated in the sample than in the general population (details in Table 1). A smaller percentage of participants had chronic diseases in the Swedish sample compared with the general population, but this was not observed for the Norwegian sample.

**Table 1:** Socio-demographic and clinical characteristics

	Sample (n = 1019)		Population^a^	Comparison^b^		
	n	%	%	χ^2^	d.f	p-value
*Norway*						
Gender						
Male	520	51.0	50.8	0.02	1	0.883
Female	499	49.0	49.2			
Age group (years)						
18–29	172	20.5	20.4	1.65	5	0.895
30–39	150	18.4	18.1			
40–49	208	17.8	17.7			
50–59	183	18.4	17.6			
60–69	181	14.3	14.4			
70–79	160	10.6	11.8			
Education						
Primary, lower secondary	270	26.5	23.7	126.99	2	< 0.001
Upper secondary	233	22.9	39.5			
Tertiary (Bachelors, Masters, PhD)	516	50.6	36.9			
Any chronic diseases	363	35.6	38.0	2.44	1	0.118
*Sweden*						
Gender						
Male	529	50.5	50.6	0.01	1	0.937
Female	519	49.5	49.4			
Age group (years)						
18–29	172	17.4	19.7	7.95	5	0.159
30–39	208	19.8	18.1			
40–49	183	17.5	16.9			
50–59	181	17.3	17.0			
60–69	154	14.7	14.3			
70–80	160	15.3	14.0			
Education						
Primary, lower secondary	100	9.5	17.6	265.02	2	< 0.001
Upper secondary	465	44.4	57.7			
Tertiary (Bachelors, Masters, PhD)	483	46.1	24.7			
Any chronic diseases	345	32.9	37.9	11.04	1	< 0.001

*d.f.* degrees of freedom

^a^Population statistics for Norway: https://www.ssb.no/en/befolkning/folketall/statistikk/befolkning

^b^Chi-square test

^c^Population statistics for Sweden: https://www.scb.se/hitta-statistik/statistik-efter-amne/befolkning/befolkningens-sammansattning/befolkningsstatistik/

### Feedback on the DCE

Both in Norway and Sweden, most participants considered the DCE as clear or very clear (Norway 59.0%, Sweden 64.0%). Only a small percentage regarded the DCE as unclear or very unclear (Norway 17.5%, Sweden 11.7%). More than one-third of the participants in Norway (37.3%) and almost half of those in Sweden (46.9%) considered the DCE difficult or very difficult, and a considerable percentage regarded it as easy or very easy (Norway 37.4%, Sweden 25.0%). For the response strategy, a large percentage of the participants considered most or all aspects of the response sets (Norway 49.3%, Sweden 43.4%), and about 25% considered only the highlighted aspects (Norway 25.5%, Sweden 25.3%). A small percentage concentrated on only a few aspects (Norway 14.4%, Sweden 19.3%) or used other strategies (Norway 10.7%, Sweden 12.1%) (see supplementary Table 2).

### EORTC QLU-C10D utility weights for Norway and Sweden

**Fig. 2:**
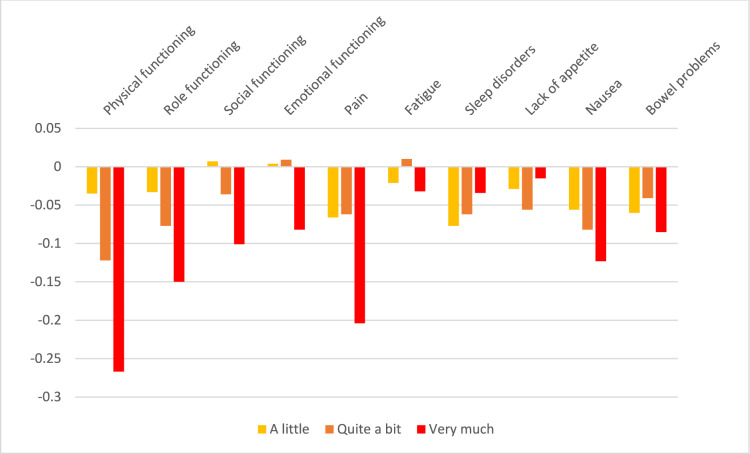
Raw utility decrements for the Norwegian version of the QLU-C10D

**Fig. 3:**
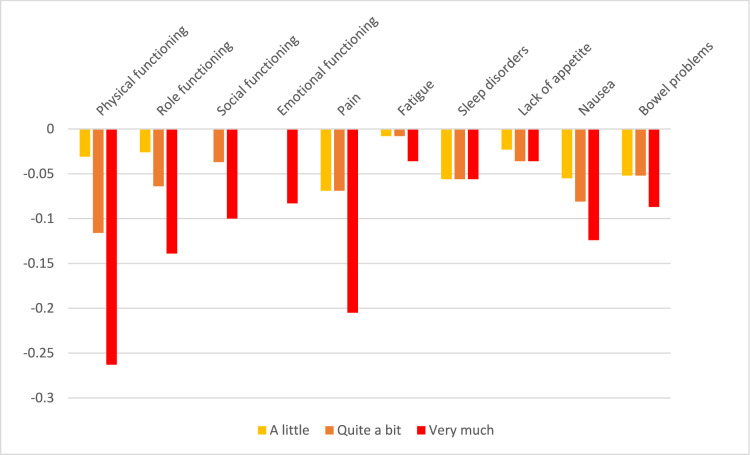
Utility decrements for the Norwegian version of the QLU-C10D with adjustment for monotonicity

**Fig. 4:**
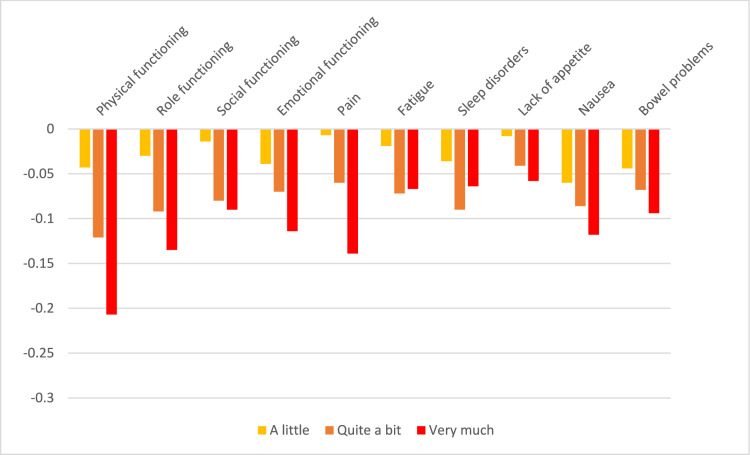
Raw utility decrements for the Swedish version of the QLU-C10D

**Fig. 5:**
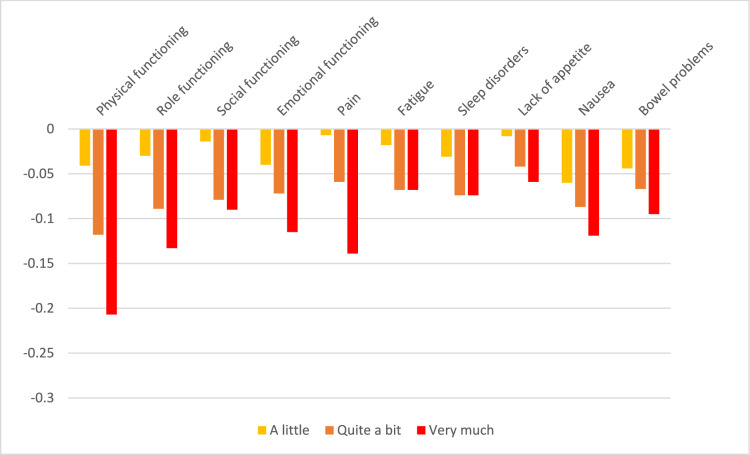
Utility decrements for the Swedish version of the QLU-C10D with adjustment for monotonicity

Descriptions of the findings of the DCE analysis are given in Table 2 and Figs. 2 and 3 for Norway and in Table 3 and Figs. 4 and 5 for Sweden.

**Table 2:** QLU-C10D utility decrements for Norway (unconstrained and adjusted for monotonicity)

Parameter	Level^a^	Unconstrained			Adjusted for monotonicity		
		Coefficient (α, β)	SE	Utility decrement (β/α)	Coefficient (α, β)	SE	Utility decrement (β/α)
Time coefficient (α)	(Linear)	0.266**	0.023		0.264**	0.022	
Attributes (β)							
Physical functioning	2	− 0.009	0.009	− 0.035	− 0.008	0.009	− 0.031
Physical functioning	3	− 0.033**	0.010	− 0.122	− 0.031**	0.010	− 0.116
Physical functioning	4	− 0.071**	0.009	− 0.267	− 0.069**	0.009	− 0.263
Role functioning	2	− 0.009	0.008	− 0.033	− 0.007	0.008	− 0.026
Role functioning	3	− 0.021*	0.009	− 0.077	− 0.017*	0.008	− 0.064
Role functioning	4	− 0.040**	0.008	− 0.150	− 0.037**	0.007	− 0.139
Social functioning	2	0.002	0.007	0.007	0.000	–	0.000
Social functioning	3	− 0.010	0.008	− 0.036	− 0.010	0.007	− 0.037
Social functioning	4	− 0.027**	0.007	− 0.101	− 0.026**	0.007	− 0.100
Emotional functioning	2	0.001	0.008	0.004	0.000	–	0.000
Emotional functioning	3	0.002	0.008	0.009	0.000	–	0.000
Emotional functioning	4	− 0.022**	0.007	− 0.082	− 0.022**	0.006	− 0.083
Pain	2	− 0.018*	0.008	− 0.066	− 0.018*	0.007	− 0.069
Pain	3	− 0.017*	0.008	− 0.062	− 0.018*	0.007	− 0.069
Pain	4	− 0.054**	0.008	− 0.204	− 0.054**	0.008	− 0.205
Fatigue	2	− 0.006	0.007	− 0.021	− 0.002	0.007	− 0.008
Fatigue	3	0.003	0.008	0.010	− 0.002	0.007	− 0.008
Fatigue	4	− 0.008	0.007	− 0.032	− 0.010	0.007	− 0.036
Sleep disorders	2	− 0.020*	0.008	− 0.077	− 0.015*	0.006	− 0.056
Sleep disorders	3	− 0.016*	0.008	− 0.062	− 0.015*	0.006	− 0.056
Sleep disorders	4	− 0.009	0.007	− 0.034	− 0.015*	0.006	− 0.056
Lack of appetite	2	− 0.008	0.007	− 0.029	− 0.006	0.007	− 0.023
Lack of appetite	3	− 0.015*	0.008	− 0.056	− 0.009	0.006	− 0.036
Lack of appetite	4	− 0.004	0.007	− 0.015	− 0.009	0.006	− 0.036
Nausea	2	− 0.015*	0.007	− 0.056	− 0.015*	0.007	− 0.055
Nausea	3	− 0.022*	0.008	− 0.082	− 0.021*	0.008	− 0.081
Nausea	4	− 0.033**	0.007	− 0.123	− 0.033**	0.007	− 0.124
Bowel problems	2	− 0.016*	0.007	− 0.060	− 0.014*	0.007	− 0.052
Bowel problems	3	− 0.011	0.008	− 0.041	− 0.014*	0.007	− 0.052
Bowel problems	4	− 0.023**	0.007	− 0.085	− 0.023**	0.007	− 0.087

Domains and levels with non-monotonicity are highlighted in bold. *p < 0.05, **p < 0.01

^a^Level 2 = a little; Level 3 = quite a bit; Level 4 = very much

*SE* standard error

**Table 3:** QLU-C10D utility decrements for Sweden (unconstrained and adjusted for monotonicity)

Parameter	Level^a^	Unconstrained			Adjusted for monotonicity		
		Coefficient (α, β)	SE	Utility decrement (β/α)	Coefficient (α, β)	SE	Utility decrement (β/α)
Time coefficient (α)	(Linear)	0.533**	0.033		0.528**	0.033	
Attributes (β)							
Physical functioning	2	− 0.023*	0.012	− 0.043	− 0.022	0.012	− 0.041
Physical functioning	3	− 0.064**	0.013	− 0.121	− 0.063**	0.013	− 0.118
Physical functioning	4	− 0.110**	0.012	− 0.207	− 0.109**	0.012	− 0.207
Role functioning	2	− 0.016	0.009	− 0.030	− 0.016	0.009	− 0.030
Role functioning	3	− 0.049**	0.010	− 0.092	− 0.047**	0.010	− 0.089
Role functioning	4	− 0.072**	0.009	− 0.135	− 0.070**	0.009	− 0.133
Social functioning	2	− 0.007	0.009	− 0.014	− 0.008	0.009	− 0.014
Social functioning	3	− 0.043**	0.010	− 0.080	− 0.042**	0.010	− 0.079
Social functioning	4	− 0.048**	0.009	− 0.090	− 0.047**	0.009	− 0.090
Emotional functioning	2	− 0.021*	0.009	− 0.039	− 0.021*	0.009	− 0.040
Emotional functioning	3	− 0.037**	0.009	− 0.070	− 0.038**	0.010	− 0.072
Emotional functioning	4	− 0.061**	0.009	− 0.114	− 0.061**	0.009	− 0.115
Pain	2	− 0.004	0.009	− 0.007	− 0.004	0.009	− 0.007
Pain	3	− 0.032**	0.010	− 0.060	− 0.031**	0.010	− 0.059
Pain	4	− 0.074**	0.009	− 0.139	− 0.073**	0.009	− 0.139
Fatigue	2	− 0.010	0.008	− 0.019	− 0.009	0.008	− 0.018
**Fatigue**	3	− 0.038**	0.009	− 0.072	− 0.036**	0.008	− 0.068
**Fatigue**	4	− 0.035**	0.008	− 0.067	− 0.036**	0.008	− 0.068
Sleep disorders	2	− 0.019*	0.009	− 0.036	− 0.016	0.009	− 0.031
**Sleep disorders**	3	− 0.048**	0.010	− 0.090	− 0.039**	0.008	− 0.074
**Sleep disorders**	4	− 0.034**	0.009	− 0.064	− 0.039**	0.008	− 0.074
Lack of appetite	2	− 0.004	0.008	− 0.008	− 0.004	0.009	− 0.008
Lack of appetite	3	− 0.022*	0.009	− 0.041	− 0.022*	0.009	− 0.042
Lack of appetite	4	− 0.031**	0.009	− 0.058	− 0.031**	0.009	− 0.059
Nausea	2	− 0.032**	0.009	− 0.060	− 0.032**	0.009	− 0.060
Nausea	3	− 0.046**	0.010	− 0.086	− 0.046**	0.010	− 0.087
Nausea	4	− 0.063**	0.009	− 0.118	− 0.063**	0.009	− 0.119
Bowel problems	2	− 0.023**	0.009	− 0.044	− 0.023**	0.009	− 0.044
Bowel problems	3	− 0.036**	0.009	− 0.068	− 0.036**	0.009	− 0.067
Bowel problems	4	− 0.050**	0.008	− 0.094	− 0.050**	0.009	− 0.095

Domains and levels with non-monotonicity are highlighted in bold. *p < 0.05, **p < 0.01

^a^Level 2 = a little; Level 3 = quite a bit; Level 4 = very much

*SE* standard error

In Norway, problems with non-monotonicity (non-increasing utility decrements with increasing level of severity) were observed for seven of the 10 domains (all domains except physical functioning, role functioning, nausea). However, none of the cases of non-monotonicity reached statistical significance. Both the uncorrected and monotonicity-adjusted utility weights are shown in Table 3 (uncorrected values on the left and corrected values on the right). In the reminder of this paper, we concentrated on the monotonicity-adjusted findings. The largest utility decrements were seen for the domain of physical functioning (decrement of − 0.263 for highest level “very much”), followed by pain (decrement − 0.205 for “very much”) and role functioning (− 0.139). Among the cancer-specific domains, nausea showed the largest utility decrement (− 0.124), but the other cancer-specific domains had small utility decrements. The lowest possible utility value with a severity level of “very much” in all domains amounted to − 0.129 and corresponded to a utility worse than death.

In Sweden, lack of monotonicity was encountered only in two domains: fatigue and sleep disorders. Again, the effects of non-monotonicity (differences between adjacent levels) were small and did not reach statistical significance. Non-monotonicity was adjusted for in the adjusted utility values (Table 3). As in Norway, the largest utility decrements were observed for physical functioning (− 0.207 for level “very much”), followed by pain (− 0.139), role functioning (− 0.133), and nausea (− 0.119). Emotional functioning also exhibited sizable utility decrements (− 0.115) (see Table 3). The lowest possible utility value for the health state with a severity of “very much” in all domains was − 0.099, which was slightly larger than the corresponding value for Norway.

Utility weights can be calculated by subtracting the decrements (we recommend to use corrected decrements) from Tables 2 and 3 from 1 (state of perfect health). For example, the utility score for the worst possible health state (severity level of “very much” for all domains) would be calculated as follows for Sweden (see also https://qol.eortc.org/eortc-qlu-c10d/):

$$ 1{-}0.207{-}0.133{-}0.090{-}0.115{-}0.139{-}0.068{-}0.074{-}0.059{-}0.119{-}0.095 = {-}0.099 $$

### Data quality checks

Extreme response patterns (always response option “A” or “B” in the DCE) were observed only in a few cases: in 1.2% of the participants from Norway and in 1.5% from Sweden. After removing the 10% fastest respondents from the sample, DCE results remained relatively stable. In Norway, the differences in utility decrements between the complete and reduced samples ranged from − 0.032 to 0.019, and the MAD was 0.011. In Sweden, the differences ranged from − 0.014 to 0.012, and the MAD was 0.006.

## Discussion

We have developed Norwegian and Swedish utility weights for the EORTC QLU-C10D from samples of the general population of these two countries. The recruited samples were representative of the Norwegian and Swedish populations in terms of age and gender. In both countries, the domains with the largest utility decrements that had the biggest impact on health utility were physical functioning, pain, and role functioning, and in the Swedish population, emotional functioning.

### The EORTC QLU-C10D and relevance for Norway and Sweden.

In Norway, the National Health Insurance is responsible for evaluating medicines issued under its scheme. Additionally, the Norwegian National System for Managed Introduction of New Health Technologies within the Specialist Health Care Services guides funding decisions for the implementation of new technologies and medicines for use in Norwegian hospitals. These entities rely on principles of priority setting, which entail the benefit criterion, resource criterion, and severity criterion. The benefit criterion is specified further, and the use of QALYs is mentioned explicitly for quantifying the benefits [1, 2, 40].

In Sweden, two main entities are responsible for health economic evaluation. Firstly, 20 directly elected county councils are responsible for providing health services to the population and setting preferences about the types of services to be offered. Secondly, the Dental and Pharmaceutical Benefits Agency is responsible for drug appraisals of the prescription and disposal of medicines. Sweden relies on cost-effectiveness analysis in health economic appraisal, but this analysis is subordinate to the other two guiding principles of human value and need and solidarity when making resource-allocation decisions [1, 4]. In general, the use of CUA and QALYs is recommended by the Dental and Pharmaceutical Benefits Agency [41].

The importance of CUA and QALYs for prioritization and evaluation in health care is emphasized in governmental documents and guidelines, such as those mentioned above, in both Norway and Sweden. Although generic utility measures such as the EQ-5D and the SF-6D are used in cancer care, our current study offers national utility weights to be used with the cancer-specific EORTC QLU-C10D. The EORTC QLU-C10D is based on the widely used EORTC QLQ-C30 questionnaire [16], which has been used in academic and pharmaceutical research for decades, and data from clinical trials that included the EORTC QLQ-C30 can be used to generate utility scores (even retrospectively) that can be used to calculate QALYs. The Norwegian and Swedish findings for studies using the EORTC QLU-C10D can be compared to the growing body of cancer-specific utility weights for this questionnaire available for different countries [23, 24, 26–31, 42].

### Utility weights in Norwegian and Swedish populations and other countries

The finding of the largest utility decrement for physical functioning from both Norway and Sweden has also been observed in other European countries such as Denmark [30], Germany [26], and Austria [31]. Overall, pain was the domain with the second-largest decrements and the largest for the symptom domains in the EORTC QLU-C10D questionnaire. This pattern has also been observed in other countries such as Denmark [30], Austria [31], Germany [26], Spain [29], France [27], and the UK [24]**.** The worst attainable health status (i.e., having the largest impairment in all domains) was − 0.129 for Norway and − 0.099 for Sweden; these values indicate that, in both countries, this health status would be rated as worse than being dead. In general, the utility decrements for the EORTC QLU-C10D in Norway and Sweden and the rank order of domains were similar to those in other European countries.

### Differences between Norway and Sweden

Normally, the Norwegian and Swedish peoples are presumed to be similar, and this appeared to be so for the variables examined in the present study. However, a few differences were identified in our study. The domain of emotional functioning had a larger utility decrement in Sweden than in Norway, and this difference suggests that emotional outcomes are more valued or considered to have a stronger impact in the Swedish population. We do not presume this to be a difference based on wording, as the translations of “depressed” are similar in Norwegian and Swedish. Similarly, a large impact of emotional function was identified in Denmark [30]. In international comparisons, only Denmark [30], France [27], the UK [24], and Australia [42] have larger utility decrements for emotional functioning than Sweden.

Pain was the second-largest utility decrement in both countries and the symptom domains with the largest decrement. The utility decrement for pain was larger in Norway than in Sweden, and the Norwegian decrement was similar to those reported in Dutch [28], French [27], and German [26] studies. This pattern was the opposite for fatigue in that a smaller utility decrement was observed in the Norwegian population than in the Swedish population. Other countries report utility decrements for the fatigue domain in between these two [26, 28, 29, 31].

In terms of monotonicity [38] of utility decrements, we found more problems with non-monotonicity for Norway (seven domains) than for Sweden (two domains). These may have resulted from the translation of the four response levels (“not at all”, “a little”, “quite a bit”, “very much”), which differs somewhat between the Norwegian and Swedish versions of the EORTC QLQ-C30 and EORTC QLU-C10D, with the wording of the Norwegian response options being a bit vaguer than the Swedish ones.

The participants from both countries included in the present study had the same age and gender distributions as the general populations in these countries [37, 38]. However, the Swedish participants had fewer chronic diseases compared with the general population [38], but this was not the case for the Norwegian participants [37]. The abovementioned differences may have influenced the external validity of the results, and the external validity may be better for the Norwegian than the Swedish findings.

### Strengths and limitations

A strength of this study is that we included 1019 Norwegian and 1048 Swedish participants who were representative of their respective general populations with regard to age and gender. The standardised methodology used in the present study is reliable [17], which enables sound comparisons between countries. One limitation of this study is that the participants are not representative of the Norwegian and Swedish population in terms of education level because the participants were more highly educated than the general population. A significantly smaller percentage of the Swedish participants had chronic diseases compared with the general population. Another limitation is that non-monotonicities were encountered; i.e., an increasing EORTC QLU-C10D domain level did not coincide with increasing utility decrements in some domains. In general, differences between adjusted and non-adjusted utility decrements were quite small (see Tables 2 and 3). These differences could be the result of respondents having problems differentiating between some adjacent levels in the DCE for some domains. However, none of these non-monotonicities reached statistical significance. Imposed constraints were used to remove non-monotonicities in the final analyses, as in all previous EORTC QLU-C10D valuations [22–24, 26–31].

Finally, we only had access to respondents who completed the whole survey. Hence we are not able to compare complete responders and partial responders, as for example, done previously [43], where only minor differences between responders and partial responders where found.

## Conclusion

We have developed utility weights for the EORTC QLU-C10D that can be used for CUA for the Norwegian and Swedish general populations. The utility decrements will help provide more targeted decision-making for cancer treatment and care in Norway and Sweden. From an international perspective, the results add to a growing body of evidence about the EORTC QLU-C10D that will be useful when making decisions about existing and new cancer treatments and supportive care interventions, and when comparing these between countries.

## Supplementary Information

Below is the link to the electronic supplementary material.

Supplementary file1 (DOCX 360 KB)
