# Supplementary material for: Norwegian and Swedish value sets for the EORTC QLU-C10D utility instrument
Source: Qual Life Res. 2024 Nov 5;34(2):429–43. doi: 10.1007/s11136-024-03824-8 (PMC11865156; doi:10.1007/s11136-024-03824-8)
Supplement: Supplementary file 1 — Supplementary file1 (DOCX 360 KB) [file 11136_2024_3824_MOESM1_ESM.docx]

**Supplementary Materials: Rohde et al. 2024**

**Supplementary table 1. Health state classification system of the QLU-C10D**

| **Dimension** | **Level** | **Stem** | **Descriptor** | **QLQ-C30 item scores** |
| --- | --- | --- | --- | --- |
| Physical functioning | 1 | You have… | No trouble taking a long walk outside the house | Item 2 (long walk) = 1 |
|  | 2 |  | No trouble taking a short walk outside the house, but at least a little trouble taking a long walk | Item 3 (short walk) = 1 AND Item 2 ≥ 2 |
|  | 3 |  | At least a little trouble taking a short walk outside the house, and at least a little trouble taking a long walk | Item 3 = 2 AND Item 2 ≥ 2 |
|  | 4 |  | Quite a bit or very much trouble taking a short walk outside the house | Item 3 ≥ 3 AND Item 2 ≥ 2 |
| Role functioning | 1 | You are limited in pursuing your work or other daily activities… | Not at all | Item 6 = 1 |
|  | 2 |  | A little | Item 6 = 2 |
|  | 3 |  | Quite a bit | Item 6 = 3 |
|  | 4 |  | Very much | Item 6 = 4 |
| Social functioning | 1 | Your physical condition or medical treatment interferes with your social or family life… | Not at all | Items 26 AND 27 = 1 |
|  | 2 |  | A little | Items 26 OR 27 = 2 |
|  | 3 |  | Quite a bit | Items 26 OR 27 = 3 |
|  | 4 |  | Very much | Items 26 OR 27 = 4 |
| Emotional functioning | 1 | You feel depressed… | Not at all | Item 24 = 1 |
|  | 2 |  | A little | Item 24 = 2 |
|  | 3 |  | Quite a bit | Item 24 = 3 |
|  | 4 |  | Very much | Item 24 = 4 |
| Pain | 1 | You have pain… | Not at all | Item 9 = 1 |
|  | 2 |  | A little | Item 9 = 2 |
|  | 3 |  | Quite a bit | Item 9 = 3 |
|  | 4 |  | Very much | Item 9 = 4 |
| Fatigue | 1 | You feel tired… | Not at all | Item 18 = 1 |
|  | 2 |  | A little | Item 18 = 2 |
|  | 3 |  | Quite a bit | Item 18 = 3 |
|  | 4 |  | Very much | Item 18 = 4 |
| Sleep | 1 | You have trouble sleeping… | Not at all | Item 11 = 1 |
|  | 2 |  | A little | Item 11 = 2 |
|  | 3 |  | Quite a bit | Item 11 = 3 |
|  | 4 |  | Very much | Item 11 = 4 |
| Appetite | 1 | You lack appetite… | Not at all | Item 13 = 1 |
|  | 2 |  | A little | Item 13 = 2 |
|  | 3 |  | Quite a bit | Item 13 = 3 |
|  | 4 |  | Very much | Item 13 = 4 |
| Nausea | 1 | You feel nauseated… | Not at all | Item 14 = 1 |
|  | 2 |  | A little | Item 14 = 2 |
|  | 3 |  | Quite a bit | Item 14 = 3 |
|  | 4 |  | Very much | Item 14 = 4 |
| Bowel problems | 1 | You… | Do not have constipation or diarrhoea at all | Items 16 AND 17 = 1 |
|  | 2 |  | Have a little constipation or diarrhoea | Items 16 OR 17 = 2 |
|  | 3 |  | Have constipation or diarrhoea quite a bit | Items 16 OR 17 = 3 |
|  | 4 |  | Have constipation or diarrhoea very much | Items 16 OR 17 = 4 |
| Duration | 1 | You will live in this health state for… | 1 year, and then die | Not applicable |
|  | 2 |  | 2 years, and then die | Not applicable |
|  | 3 |  | 5 years, and then die | Not applicable |
|  | 4 |  | 10 years, and then die | Not applicable |

**Supplementary table 2: Feedback information on the DCE**

| Variable |  | Norway (n=1019) | Sweden (n=1048) |
| --- | --- | --- | --- |
|  |  |  |  |
| Clarity of DCE | Clear/very clear | 59.0% | 64.0% |
|  | Unclear/very unclear | 17.5% | 11.7% |
|  |  |  |  |
| Difficulty of DCE | Easy/very easy | 37.4% | 25.0% |
|  | Difficult/very difficult | 37.3% | 46.9% |
|  |  |  |  |
| Strategy used | Considered all aspects | 24.3% | 13.0% |
|  | Considered most aspects | 25.0% | 30.4% |
|  | Highlighted aspects | 25.5% | 25.3% |
|  | Only a few aspects | 14.4% | 19.3% |
|  | Other strategy/ no strategy | 10.7% | 12.1% |

Supplementary Figure 1: English language example screenshot from the DCE survey.


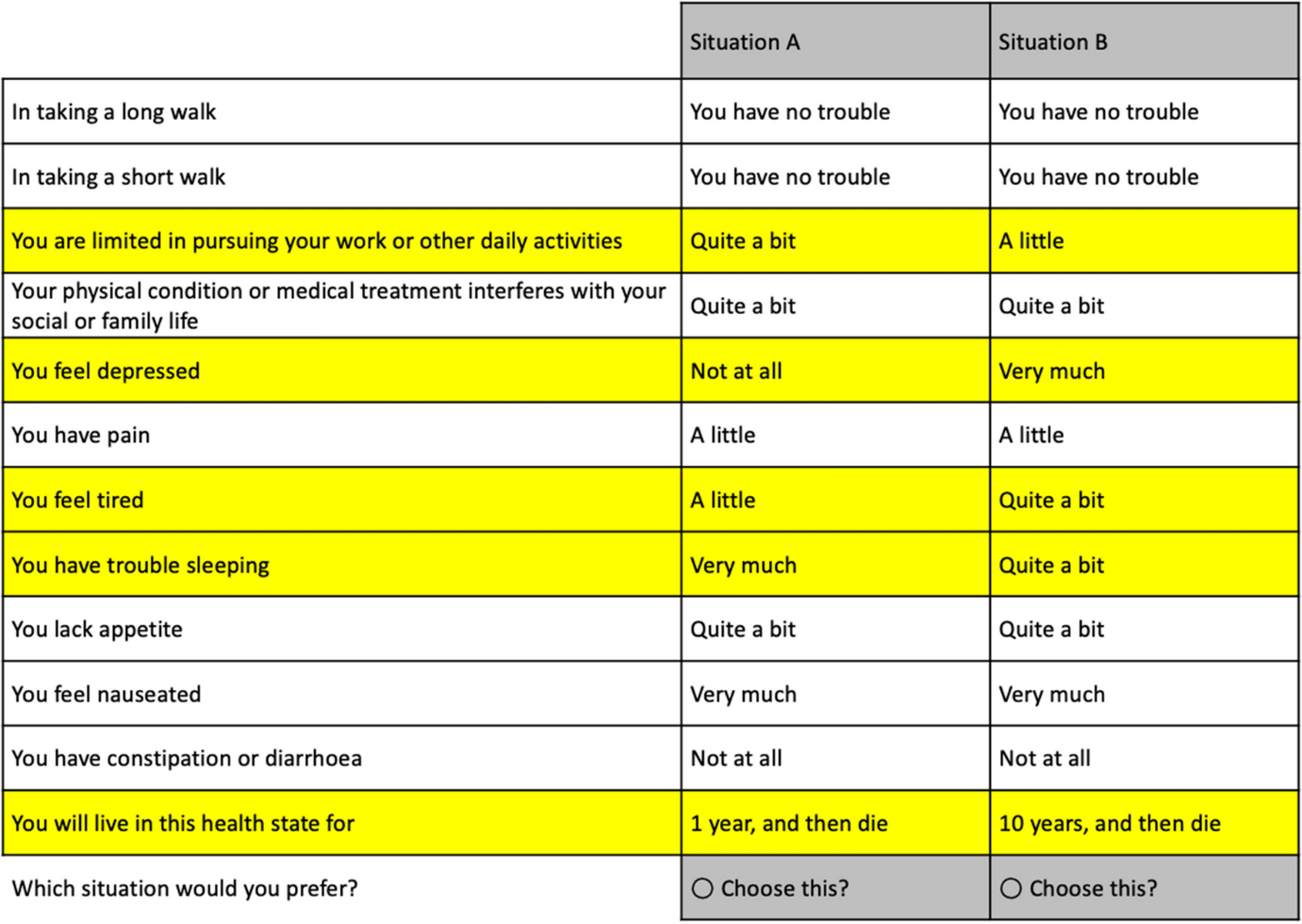


The task required participants to choose between two QLU-C10D health states, labeled as 'Situation A' and 'Situation B', each with a specified duration. In the QLU-C10D valuation methods experiment, choice sets were formatted according to participant preference, with differences between the situations highlighted in yellow. Due to the complexity of the descriptors for levels 2 and 3 of the Physical Functioning dimension, the survey separated the items 'long walk' and 'short walk' to enhance understanding. For DCE design and data analysis, the Physical Functioning dimension was treated as a single four-level dimension.

With over a million possible health states in the QLU-C10D classification system, a designed experiment selected 960 choice sets to maximize statistical efficiency for estimating utility model parameters. The survey's DCE component incorporated two levels of randomization: each respondent was randomly allocated 16 out of the 960 choice sets, and within each set, the assignment of Situation A or B was randomized to avoid ordering bias. Previous research demonstrated that presenting the dimensions in a consistent order does not systematically bias utility weights for the QLU-C10D [1, 2]

1. King MT, Viney R, Simon Pickard A, Rowen D, Aaronson NK, Brazier JE, Cella D, Costa DSJ, Fayers PM, Kemmler G, et al: Australian Utility Weights for the EORTC QLU-C10D, a Multi-Attribute Utility Instrument Derived from the Cancer-Specific Quality of Life Questionnaire, EORTC QLQ-C30. *Pharmacoeconomics* 2018, 36**:**225-238. <https://doi.org/10.1007/s40273-017-0582-5>

2. Norman R, Kemmler G, Viney R, Pickard AS, Gamper E, Holzner B, Nerich V, King M: Order of Presentation of Dimensions Does Not Systematically Bias Utility Weights from a Discrete Choice Experiment. *Value Health* 2016, 19**:**1033-1038. <https://doi.org/10.1016/j.jval.2016.07.003>
